# Metabolic gene therapy in a canine with pulmonary hypertension secondary to degenerative mitral valve disease

**DOI:** 10.3389/fvets.2024.1415030

**Published:** 2024-09-23

**Authors:** Michael G. Katz, Dan G. Ohad, Philip Putter, Nataly Shtraizent, Ehud Shahar, Smadar Tal, Efrat Eliyahu

**Affiliations:** ^1^Department of Genetics and Genomic Sciences, Icahn School of Medicine at Mount Sinai, New York, NY, United States; ^2^Department of Pediatric Cardiac Surgery, Icahn School of Medicine at Mount Sinai, New York, NY, United States; ^3^Department of Cardiology, Veterinary Teaching Hospital of the Koret School of Veterinary Medicine, The Hebrew University of Jerusalem, Rehovot, Israel; ^4^Spot On Veterinary Hospital, Stamford, CT, United States; ^5^Senex, New York, NY, United States; ^6^Frezent Biological Solutions, New York, NY, United States; ^7^Department of Biotechnology, Tel-Hai College, Kiryat Shmona, Israel; ^8^Department of Nutrition and Natural Products, Migal-Galilee Research Institute, Kiryat Shmona, Israel; ^9^Department of Veterinary Neonatology, Veterinary Teaching Hospital of the Koret School of Veterinary Medicine, The Hebrew University of Jerusalem, Rehovot, Israel; ^10^Department of Animal Sciences, Tel-Hai College, Qiryat Shemona, Israel; ^11^Icahn Genomics Institute, Icahn School of Medicine at Mount Sinai, New York, NY, United States

**Keywords:** degenerative mitral valve disease, pulmonary hypertension, gene therapy, sphingolipids metabolism, canine cardiovascular disease

## Abstract

Myxomatous mitral valve disease (MMVD) stands out as the most prevalent acquired canine heart disease. Its occurrence can reach up to 40% in small breed dogs and escalates in geriatric canine populations. MMVD leads to thickening and incomplete coaptation of valve leaflets during systole, resulting in secondary mitral valve regurgitation. Serious complications may arise concurrently with the worsening of mitral valve regurgitation, including left-and right-sided congestive heart failure, and pulmonary hypertension (PH). Ultimately, the PH progression might contribute to the patient’s demise or to the owner’s decision of euthanasia. Most currently available FDA-approved therapies for PH are costly and aim to address the imbalance between vasoconstriction and vasodilation to restore endothelial cell function. However, none of these medications impact the molecular dysfunction of cells or impede the advancement of pulmonary vascular and right ventricular remodeling. Recent evidence has showcased successful gene therapy approaches in laboratory animal models of PH. In this manuscript, we summarize the latest advancements in gene therapy for the treatment of PH in animals. The manuscript incorporates original data showcasing sample presentations, along with non-invasive hemodynamic assessments. Our findings demonstrate that the use of metabolic gene therapy, combining synthetic adeno-associated virus with acid ceramidase, has the potential to significantly reduce the need for drug treatment and improve spontaneously occurring PH in dogs.

## 1 Introduction

Myxomatous mitral valve disease (MMVD) stands out as the most prevalent acquired canine heart disease accounting for approximately 75% of heart disease cases seen in veterinary practices in the USA (1). It is typically characterized by chronic valve leaflet and chordal degeneration, leading to thickening and incomplete coaptation of valve leaflets during systole, resulting in secondary mitral regurgitation (MR). At least 30% of the patients develop a similar process in their tricuspid valve apparatus, leading to tricuspid valve regurgitation (TR) as well. This results in progressive volume overload-related remodeling and mostly dilation of the left/right atrium and ventricle (2). Serious complications may arise concurrently with the worsening of mitral valve regurgitation, including left and right-sided congestive heart failure, often preceded by mild-to-severe pulmonary post and precapillary hypertension (PH) (3). This secondary complication is common in dogs affected with MMVD, characterized by an abnormal increase in pulmonary arterial pressure (PAP) and is a poor prognostic indicator (4). Until recently, only palliative pharmacotherapy could be offered for secondary PH in these patients, through phosphodiesterase-5 inhibition using sildenafil or tadalafil (5).

The last two decades have seen a significant improvement in pharmacotherapeutic options available to PH in human patients as well. However, like canine treatment modalities, these agents remain merely palliative and come with several caveats. Acute vasodilator therapy using calcium channel blockade, is highly efficacious in only a small sub-population of patients of which only very few demonstrate vasoreactivity. Insights into the pathophysiology of PH have implicated three pathways in the development and progression of disease: the prostacyclin, nitric oxide and endothelin pathways. Each of these has since become a therapeutic target and subsequent treatments have been developed.

Although explored in the treatment of PH, gene-based therapy has not been substantively pursued, perhaps due to the inherent challenges of the technology at the present time, which include efficient vector design and delivery, potential risks of insertional mutagenesis, duration of effect, and precise targeting of the key cellular sites of disease.

## 2 Pulmonary hypertension in dogs

### 2.1 Prevalence

While the true incidence of PH in dogs with chronic left-sided heart failure is unknown, one study of adult-onset valvular disease found the prevalence of detected PH to be 14 per cent and the prevalence of PH increased with increasing MV regurgitant jet size (6). In another study of dogs with adult-onset MMVD, 31 per cent of dogs had PH (7). Left-sided cardiac dysfunction appears to be a common cause of PH diagnosed clinically in dogs; combined data from three recent retrospective studies of PH in dogs suggest that approximately 40 percent of patients with diagnosed PH are thought to have increased LA pressure as the underlying etiology (8–10) while another study of dogs with tricuspid insufficiency found that 75 percent of dogs with equivocal or elevated PAP had left-sided heart disease noted as the probable cause (11).

### 2.2 Pathophysiology

Moderate to severe MR induces compensatory left ventricular (LV) and left atrial (LA) dilation in the initial phase, but over time, leads to LV systolic and diastolic dysfunction, reduced LA compliance, and elevated LA pressure in the decompensated phase (3). Long-standing passive PH resulting from venous congestion can lead to structural changes in the distal pulmonary arterioles and endothelial injury with vascular functional abnormalities, which can result in reactive pre-capillary pulmonary arterial hypertension. Sustained elevation of right ventricular (RV) afterload causes or exacerbates existing tricuspid regurgitation (TR), impairment of RV function, and rise in RA pressure, which, in turn, can complicate congestive heart failure in severe MR by causing renal dysfunction as a result of systemic venous congestion and expansion of intravascular volume leading to a vicious cycle of worsening MR, pulmonary venous (post capillary) and arteriolar (pre-capillary) congestion and hypertension, namely PH (3).

## 3 Gene therapy in animals with pulmonary hypertension

**Figure 1:**
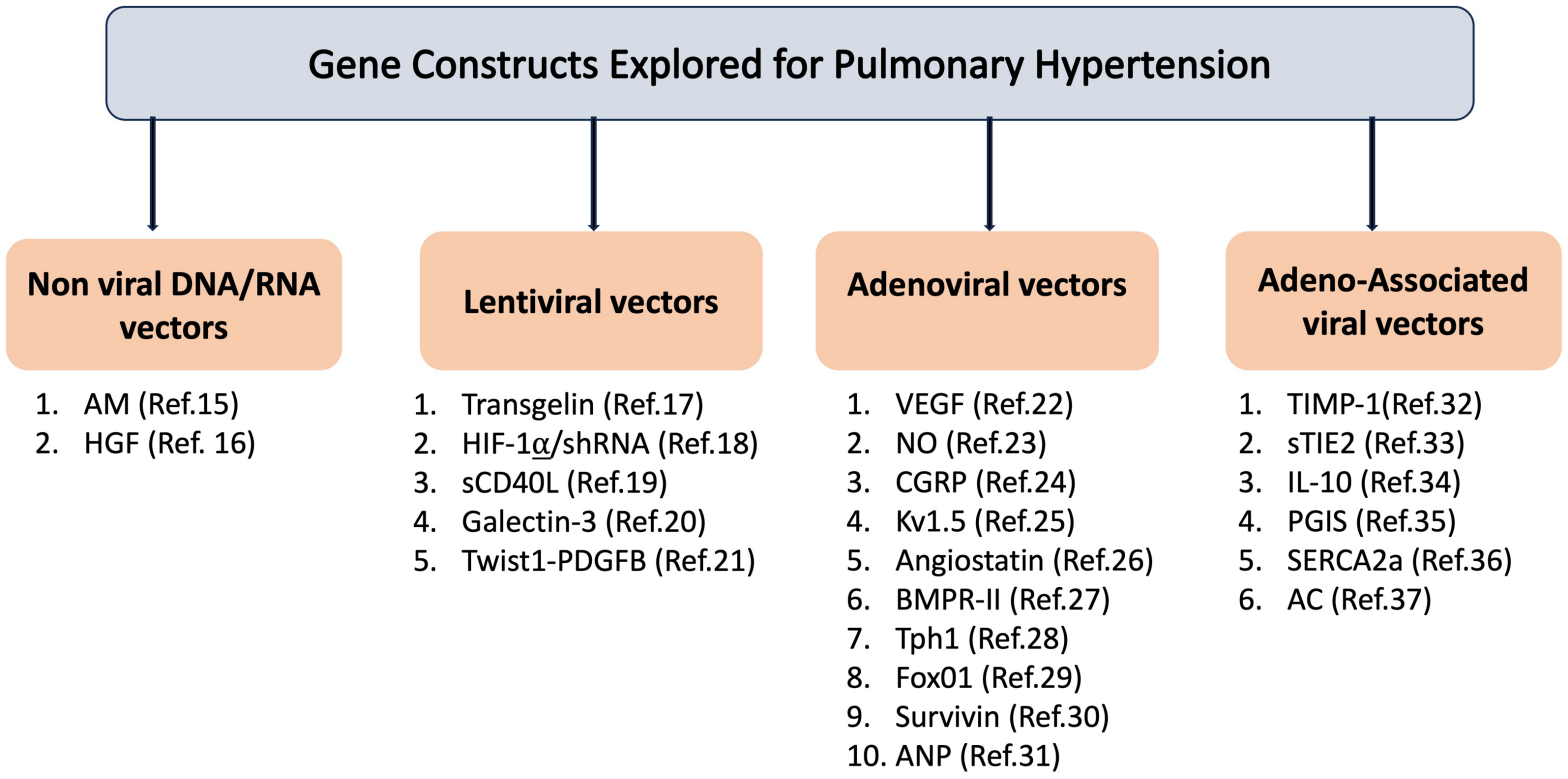
Gene construct explored for pulmonary hypertension. AM: Adrenomedullin; HGF: Hepatocyte Growth Factor; HIF-1α/shRNA: Hypoxia-Inducible Factor 1-alpha/short hairpin RNA; sCD40L: Soluble CD40 Ligand; Twist1-PDGFB: Twist1-Platelet-Derived Growth Factor Subunit B; VEGF: Vascular Endothelial Growth Factor; NO: Nitric Oxide; CGRP: Calcitonin Gene-Related Peptide; Kv1.5: Voltage-Gated Potassium Channel Subunit Kv1.5; BMPR-II: Bone Morphogenetic Protein Receptor Type II; Tph1: Tryptophan Hydroxylase1; FoxO1: Forkhead Box Protein O1; ANP: Atrial Natriuretic Peptide; TIMP-1: Tissue Inhibitor of Metalloproteinases 1; sTIE2: Soluble TIE2; IL-10: Interleukin-10; PGIS: Prostacyclin Synthase; SERCA2a: Sarco/Endoplasmic Reticulum Calcium ATPase 2a; AC: Acid Ceramidase.

The decoding of the mammalian genome has led to the discovery of new mechanisms specific to pulmonary hypertension and identification of new targets for therapeutic intervention. Defective genes due to mutation or dysregulation of gene expression may contribute to PH by promoting cell proliferation, senescence, inflammation, apoptosis, damage of endothelial cells, and altered electrolyte homeostasis. Gene-based therapy allows the delivery of nucleic acids into mutated or failing cells and has the ability to selectively target intracellular disease-specific manifestations and molecular signaling pathways in the setting of cellular dysfunction. A large body of evidence has demonstrated that restoration or down-regulation of gene expression by therapeutic delivery of targeted-genes may reduce or inhibit the PH progression (12–37) (Figure 1).

Cloning approaches have revealed that the pathogenesis of pulmonary hypertension involves a set of susceptible genes, notably mutations in genes related to the transforming growth factor-*β* (TGF-β) pathway, such as bone morphogenic protein receptor type II (BMPR-II), activin receptor-like 1 (ACVRL1), and endoglin (ENG). Gene microarray analysis underscores the importance of genetic predisposition in the development of PH.

Moreover, emerging evidence suggest that gene delivery into the lungs, with the aim of correcting deficiencies or mutations in these genes, presents a promising avenue for treating this disease (12). While significant progress has been documented in this field, particularly in gene transfer into the lungs, challenges persist concerning the design of efficient vectors, the risks of insertional mutagenesis, the duration of therapeutic effects, and the precise targeting of critical cellular sites implicated in PH (12). Here in we are suggesting a disease-modifying therapy, incorporating gene manipulation of sphingolipid metabolism. By targeting key enzymes, acid ceramidase (AC) and regulatory elements involved in sphingolipid metabolism, gene therapy aims to restore balance, mitigate ceramide accumulation, and alleviate downstream pathological effects of PH. Sphingolipids mainly, ceramide is structural and functional components in many cell pathways. The central role of ceramide in oxidative stress, initiating apoptosis, senescence, cell growth, inflammation and proliferation led to an investigation of its role in pulmonary hypertension. AC can induce both: reduce the apoptotic/senescence effect by hydrolyzing ceramide and increase the sphingosine-1-phosphate levels initiating the survival effect in PH. Deficiency in AC has been linked to significant impairments in lung function, including decreased compliance and increased airway resistance (38). Additionally, AC deficiency leads to chronic lung injury characterized by inflammation, increased vascular permeability, and alterations in surfactant activity (38). Given prior evidence demonstrating elevated ceramide levels in the lungs of patients with idiopathic PH, it is plausible to consider AC as a significant molecular factor required for down-regulating ceramide levels in PH.

## 4 Clinical presentation as a proof-of-concept for using metabolic gene therapy in dog’s pulmonary hypertension

### 4.1 Proof-of-concept

An 11-year-old, female spayed, mixed breed dog, weighing 5.6 kg, was referred to a university veterinary hospital for a cardiac evaluation due to dyspnea on exertion, cyanosis during excitement, and fatigue after exercise. The owner also reported progressively frequent seizure-like episodes characterized by vocalization, neck and foreleg extension, and loss of consciousness, typically occurring following excitement. Reduced appetite and reluctance to move were also noted.

**Figure 2:**
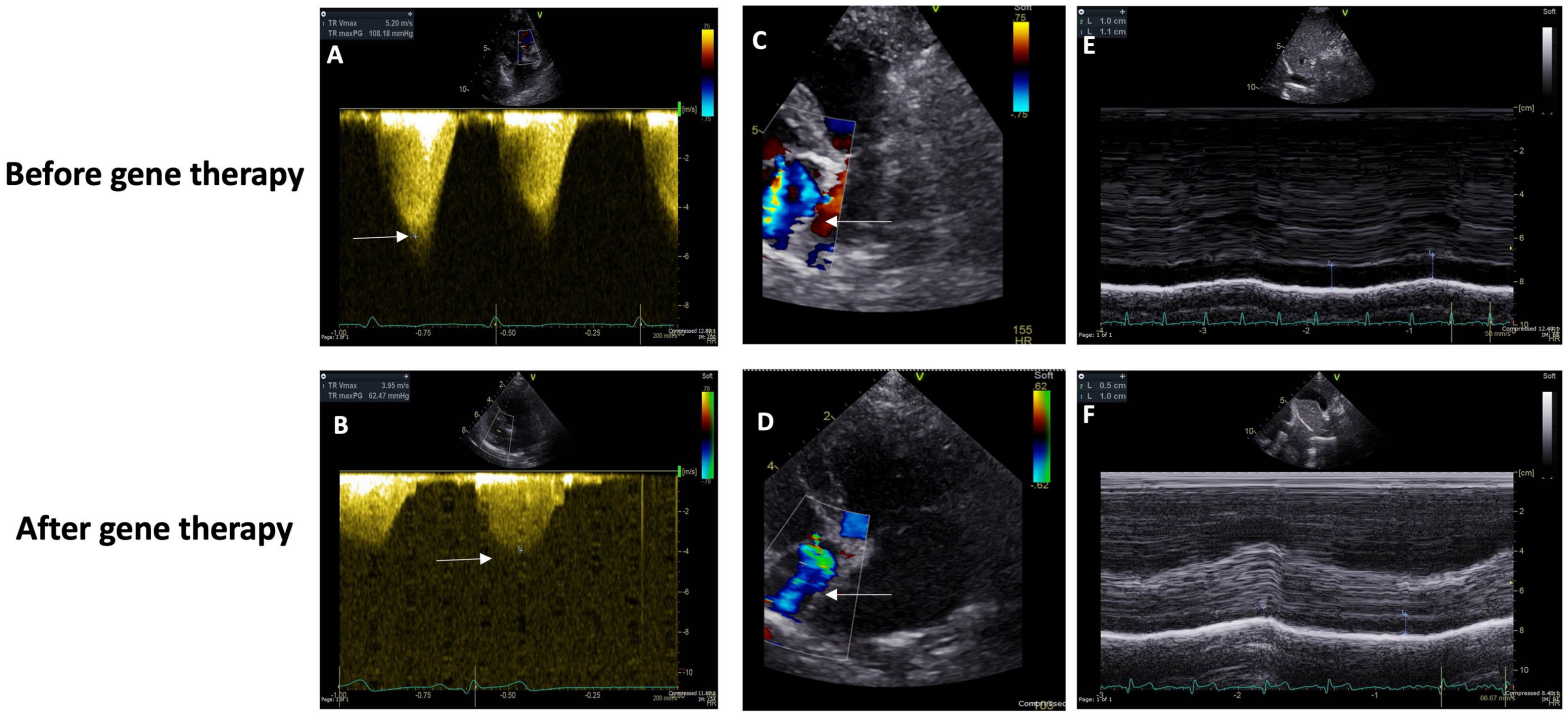
**(A,C)** Baseline Doppler echocardiographic imaging (using an S5 phased array transducer, Vivid E95, General Electric) of tricuspid valve insufficiency from the left parasternal, apical four-chamber view. Color Doppler flow **(C)** and continuous wave spectral Doppler measurement of the systolic pressure gradient (108.2 mmHg, approximately 4.3 times the expected normal) across the valve **(A)**. **(D,B)** Doppler echocardiographic imaging (using an S5 phased array transducer, Vivid E95, General Electric) of tricuspid valve insufficiency from the left parasternal, apical four-chamber view, 146 days following baseline imaging. Color Doppler flow **(C)** and continuous wave spectral Doppler measurement of the systolic pressure gradient (62.5 mmHg, approximately 2.5 times the expected normal) across the valve **(A)**. **(E)** Before gene therapy Caudal (“Posterior”) Vena Cava Diameter. **(F)** After gene therapy Caudal (“Posterior”) Vena Cava Diameter.

Upon physical examination, a systolic murmur graded IV/VI along with a Grade II/VI diastolic murmur were identified over the pulmonic valve area. An electrocardiogram revealed right axis deviation (mean electrical axis, 159°). Thoracic radiography revealed severe cardiomegaly, evidenced by a substantial increase in sternal contact with the cardiac silhouette and an elevated cardiac apex, both common signs of right-sided cardiomegaly. Echocardiography unveiled left atrial enlargement based on an increased left atrium to aortic ratio (LA/Ao) of 2.25 (normal is ≤1.5) and a mildly elevated peak tricuspid regurgitation pressure gradient (TRPG) of 42 mmHg (normal is ~25 mmHg) (Figure 2).

The patient commenced oral medical therapy according to current guidelines, including pimobendan (3.75 mg/day), a heart medication to treat dogs with congestive heart failure caused by valvular insufficiency. Tadalafil (6.25 mg every 48 h), a vasodilator to decrease high pressure in pulmonary arteries. Enalapril (6.25 mg/day), an angiotensin-converting enzyme to treat heart failure. And Spironolactone (18.75 mg/day), a diuretic to increase the amount of urine produced and excreted from the body.

Four months later, re-evaluation revealed multiple syncope episodes attributed to a significantly higher (and by now, severe) TRPG of 108 mmHg, reflecting progressive pulmonary arterial hypertension.

The treatment plan was adjusted, with an increased pimobendan dosage, introduction of furosemide, and addition of tadalafil at 6.25 mg/day. Echocardiographic findings at that time indicated moderate-to-severe degenerative valve disease affecting both mitral and tricuspid valves (Stage C of MMVD classification) accompanied by severe pulmonary hypertension (Group 2).

Nine months after the pulmonary hypertension diagnosis and following the receipt of ethical approval from the Hebrew University IACUC, the animal received 3 mL of gene construct by endotracheal delivery of the acid ceramidase gene regulated by a synthetic adeno-associated viral vector (AC/Anc80L65) at a dose of 2.5×10^12 viral genome particles. Several approaches to optimize current AAV vectors seek to improve transduction via different routes of administration. To develop synthetic distinct AAVs it was reported AAV with reconstructed viral capsids along with evolutionary lineage of AAV (39). In our previous study we found that levels of main classes of ceramides in the lung were significantly higher in PH compares to sham. AC gene overexpression was able to significantly decrease this level. Also, AC was able to decrease expression of senescence markers (37). Measuring AC activity in the lungs is possible using qPCR or Western blotting. However, this requires a biopsy, and obtaining permission for this procedure is difficult due to ethical concerns.

Two weeks post-gene therapy, a significant improvement was reported in the patient’s overall well-being, albeit experiencing some coughing and a single pre-syncope spell without vocalization, limb extension or complete loss of consciousness. Four weeks later, a follow-up examination revealed stabilized sleeping respiratory rate and a much-decreased frequency and duration of syncope spells. Tadalafil administration was minimized again to 6.25 mg every 48 h, taking care to avoid dosing in the mornings of echocardiographic imaging appointments.

The patient’s condition continued to improve over subsequent visits, with a further decrease in pre-syncope frequency, stable and normal sleeping respiratory rate, and an increased body weight to 7.8 kg. Unfortunately, despite initial and long-lasting improvement, the patient’s demise (during sleep) was reported, 3.28 years, following her initial diagnosis and 2.75 years following the documentation of severe PH at 80 mmHg.

In her last echocardiographic imaging, 1.5 years after, her tadalafil and diuretic dosages were decreased to a minimum and her PH severity was much improved with a TRPG o 50 mmHg. Overall, she survived 3.39 years after the diagnosis of PH was established and 2.55 years post-gene therapy (Table 1).

**Table 1:** Summary of the clinical data and treatments.

		Before gene therapy	4 month post gene therapy	6 month post gene therapy	9 month post gene therapy	16 month post gene therapy
Physical and clinical findings	Body weight	6.86	6.5	6.5	7.8	7.4
	Syncope	Multiple, frequent	Pre-syncope 1 time	Pre-syncope 2 times	No	No
	Seizure activity	Yes	No	No	No	No
	Neck/foreleg extension	Yes	No	No	No	No
	Systolic mitral insufficiency murmur grade	IV/VI	IV/VI	IV/VI	IV/VI	IV/VI
*Thoracic Radiography	**VHS	12.5 vb	11.9 vb	N/A	N/A	N/A
	***VLAS	3.0 vb	2.0 vb	N/A	N/A	N/A
Echocardiography	La/Ao	3.4/1.2 = 2.83	3.3/1.2 = 2.75	3.1/1.0 = 3.1	2.8/1.2 = 2.33	2.42/1.1 = 2.2
	TRPG (mmHg)	108	100.5	78.36	63.3	50
Treatment doses	Pimobendan	0.55 mg/day	0.55 mg/day	0.55 mg/day	0.55 mg/day	0.55 mg/day
	Tadalafil	6.25 mg/day	3.125 mg/day	1.56 mg/day	1.56 mg/day	None
	Enalapril	7.5 mg/day	7.5 mg/day	7.5 mg/day	7.5 mg/day	7.5 mg/day
	Spironolactone	6.25 mg/day	6.25 mg/day	6.25 mg/day	6.25 mg/day	3.125 mg/day

*A diagnostic imaging technique used to assess the thorax (chest) and lungs. **Vertebral Heart Score. ***Vertebral Left Atrial Size.

### 4.2 Immunogenicity and molecular assessment

Immunogenicity is a critical consideration in gene therapy, as the introduction of foreign genetic material can prompt immune responses, potentially reducing treatment effectiveness and leading to adverse reactions. Factors such as vector type, delivery method, and transgene characteristics can influence immunogenicity. Addressing these concerns is vital for improving the safety and efficacy of gene therapy interventions. Our immunogenicity studies demonstrate a notable rise in IgG levels toward the AAV ANC80 virus after 4 weeks, with no corresponding increase observed against the AC protein (Supplementary Figure S1). This trend, along with the lack of antibodies against AC, suggests a reduced likelihood of side effects associated with immune responses.

## 5 Discussion

Research involving client-owned dogs with spontaneously occurring disease has been extensively conducted in the field of gene therapy for monogenic diseases, demonstrating significant advantages over other animal models. Key benefits of studying such dogs include the naturally occurring incidence of causative mutations, access to comprehensive pedigree data, and the ability to conduct long-term follow-up examinations on medically treated dogs. The significance of dogs in clinical research has been underscored by the dedicated focus on “Canine Genetics” (40).

A successful gene therapy approach has been documented for canine X-linked retinitis pigmentosa. Researchers administered an AAV vector containing cDNA encoding a functional fragment of the human RPGR gene. This treatment led to a sustained preservation of retinal function, lasting for over 6.5 years (41). Additionally, canine gene therapy has shown promise in treating lysosomal storage disease (42), X-linked combined immunodeficiency (43), Duchenne muscular dystrophy (44), and various inherited bleeding disorders (45).

Existing therapies cannot treat PH and RV dysfunction and can only improve the patient symptoms without stopping the disease progression.

In our study, we are suggesting a disease-modifying therapy, incorporating gene manipulation of sphingolipid metabolism, using gene therapy. Investigations into sphingolipids have primarily centered on their roles in the proliferation and trans-differentiation of alveolar epithelial cells (46). AC serves as the principal enzyme responsible for hydrolyzing ceramide to produce free fatty acids and sphingosine, thereby mitigating the apoptotic/senescence effect.

As the proof-of-concept case, we utilized a novel synthetic AAV vector that avoids inducing toxic and inflammatory effects while facilitating efficient and early-onset expression (47). Recently, pulmonary-specific drug delivery via inhalation devices has emerged as an appealing therapeutic approach. Airway delivery offers lower endonuclease activities compared to intravascular routes, minimizing the degradation of DNA and RNA molecules, and can mitigate systemic adverse effects (48). The advantages of this gene transfer route include rapid onset of action, high local gene concentration, direct delivery to target lung tissue, and prolonged gene construct lifetime in the lung, rather than rapid metabolism and excretion (49).

Median survival times for stage C of PH in dogs are 491 days. Of the six variables used as predictors in the univariate analysis in survival, ACVIM stage C, the presence of PH, left atrium to aortic root ratio (LA/Ao) > 1.7, normalized left ventricular end-diastolic diameter (LVEDdN) > 1.73, and peak regurgitation pressure gradient (TRPG) > 55 mmHg were associated with worse outcomes. The presence of TRPG >55 mmHg and LA/Ao > 1.7 remained significant predictors of poor outcome. However, only left-atrial enlargement and a TRPG >55 mmHg were predictors of death in the multivariate analysis (3). This means that the patient reported herein survived more than twice the reported mean time while receiving only minimal doses of anti-PH medications.

## 6 Conclusion

Gene therapy in dogs represents a promising and rapidly advancing domain within contemporary medicine, especially in the treatment of cardiovascular diseases such as pulmonary hypertension. Significant efforts in gene therapy have been directed toward addressing safety and ethical concerns (50). The disease course and apparent response to gene therapy in the patient reported inhere demonstrates, for the first time, a highly promising outcome. Ongoing investigations are focused on restricting collateral organ biodistribution, targeting specific cells, and enhancing transduction efficiency.

## 7 Limitation

This project represents perspective single case report, serving as a proof of concept that our gene therapy can improve clinical outcomes and enhance long-term survival. However, the findings are limited by the small sample size. Future steps should involve conducting studies with larger sample sizes, implementing blinded clinical trials to reduce bias, and performing more extensive testing to validate the efficacy and safety of the gene therapy across diverse patient groups.

## Data Availability

The raw data supporting the conclusions of this article will be made available by the authors, without undue reservation.

## Supplementary material

SUPPLEMENTARY FIGURE S1 IgG antibody levels detected following treatment with gene therapy. Antibody levels against Acid ceramidase (AC; red line) and AAV strain ANC80 (blue line) were measured by Enzyme linked immunosorbent assay. ANC80 or AC were bound to plates diluted 1,000-fold, the dog’s sera were added to plates then specific antibodies were detected with anti-Canine IgG (H+L)-peroxidase. TMB substrate was added, and absorbance was measured at 650 nm. Results presented are subtracted by a background of negative sera from a non-treated dog. *****p* < 0.0001.
