# Supplementary material for: Metabolic gene therapy in a canine with pulmonary hypertension secondary to degenerative mitral valve disease
Source: Front Vet Sci. 2024 Sep 23;11:1415030. doi: 10.3389/fvets.2024.1415030 (PMC11457017; doi:10.3389/fvets.2024.1415030)
Supplement: SUPPLEMENTARY FIGURE S1 — IgG antibody levels detected following treatment with gene therapy. Antibody levels against Acid ceramidase (AC; red line) and AAV strain ANC80 (blue line) were measured by Enzyme linked immunosorbent assay. ANC80 or AC were bound to plates diluted 1,000-fold, the dog’s sera were added to plates then specific antibodies were detected with anti-Canine IgG (H+L)-peroxidase. TMB substrate was added, and absorbance was measured at 650 nm. Results presented are subtracted by a background of negative sera from a non-treated dog. ****p < 0.0001. [file Data_Sheet_1.docx]

**Supplementary methods for immunogenicity**

**Enzyme linked immunosorbent assay (ELISA).**

Acid ceramidase protein or AAV strain ANC80 were diluted 1000-fold in ELISA binding buffer (0.015M sodium carbonate pH 9.6), plated in 100μl volume onto ELISA plates (Thermo) and incubated overnight at 4°C. Following blocking in 37°C with 200 μl of blocking buffer (5% skim milk and 0.05% tween-20 in PBS), Mango's sera which were pre-diluted 1000-fold in dilution buffer (PBS added with 0.05% tween-20 (PBST)), were added to plates in 100 μl volume and incubated for 1 hour. Specific antibody levels were detected by further incubating plates (1hour, 37°C) with 100 μl of Goat anti-Canine IgG (H+L)–peroxidase (Invitrogen). The substrate 3,3′,5,5′-Tetramethylbenzidine (TMB) was added to plates and absorption was detected at 650nm in a plate reader (TECAN). Plates were washed 3 times with PBST after each incubation step.
