# Supplementary figures and images for: Metabolic gene therapy in a canine with pulmonary hypertension secondary to degenerative mitral valve disease
Source: Front Vet Sci. 2024 Sep 23;11:1415030. doi: 10.3389/fvets.2024.1415030 (PMC11457017; doi:10.3389/fvets.2024.1415030)

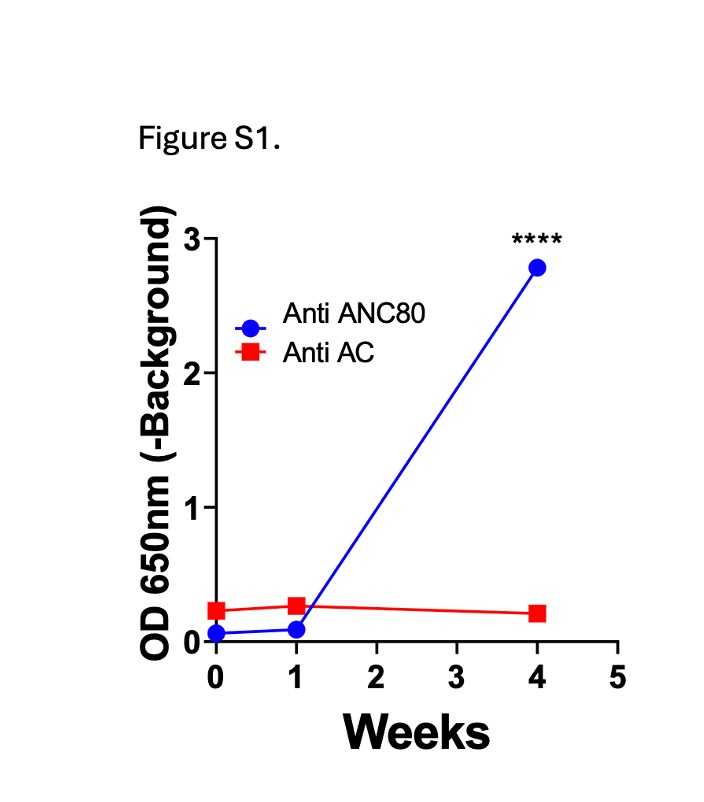

Supplement: Supplementary file 2 [file Image_1.JPEG]
